# Integrative analysis of the transcriptome profiles observed in type 1, type 2 and gestational diabetes mellitus reveals the role of inflammation

**DOI:** 10.1186/1755-8794-7-28

**Published:** 2014-05-23

**Authors:** Adriane F Evangelista, Cristhianna VA Collares, Danilo J Xavier, Claudia Macedo, Fernanda S Manoel-Caetano, Diane M Rassi, Maria C Foss-Freitas, Milton C Foss, Elza T Sakamoto-Hojo, Catherine Nguyen, Denis Puthier, Geraldo A Passos, Eduardo A Donadi

**Affiliations:** 1 Molecular Immunogenetics Group, Department of Genetics, Faculty of Medicine of Ribeirão Preto, University of São Paulo (USP), 14049-900 Ribeirão Preto, SP, Brazil; 2 Division Clinical Immunology, Faculty of Medicine of Ribeirão Preto, (USP), 14049-900 Ribeirão Preto, SP, Brazil; 3 Department of Biology, Faculty of Philosophy, Sciences and Letters, (USP), 14040-900 Ribeirão Preto, SP, Brazil; 4 INSERM U1090, TAGC, Aix-Marseille Université IFR137, 13100 Marseille, France; 5 Disciplines of Genetics and Molecular Biology, Department of Morphology, Physiology and Basic Pathology, School of Dentistry of Ribeirão Preto, USP, 14040-904 Ribeirão Preto, SP, Brazil

**Keywords:** Type 1 diabetes, Type 2 diabetes, Gestational diabetes, Module map, Immune cell signatures, Transcriptome analysis

## Abstract

**Background:**

Type 1 diabetes (T1D) is an autoimmune disease, while type 2 (T2D) and gestational diabetes (GDM) are considered metabolic disturbances. In a previous study evaluating the transcript profiling of peripheral mononuclear blood cells obtained from T1D, T2D and GDM patients we showed that the gene profile of T1D patients was closer to GDM than to T2D. To understand the influence of demographical, clinical, laboratory, pathogenetic and treatment features on the diabetes transcript profiling, we performed an analysis integrating these features with the gene expression profiles of the annotated genes included in databases containing information regarding GWAS and immune cell expression signatures.

**Methods:**

Samples from 56 (19 T1D, 20 T2D, and 17 GDM) patients were hybridized to whole genome one-color Agilent 4x44k microarrays. Non-informative genes were filtered by partitioning, and differentially expressed genes were obtained by rank product analysis. Functional analyses were carried out using the DAVID database, and module maps were constructed using the Genomica tool.

**Results:**

The functional analyses were able to discriminate between T1D and GDM patients based on genes involved in inflammation. Module maps of differentially expressed genes revealed that modulated genes: i) exhibited transcription profiles typical of macrophage and dendritic cells; ii) had been previously associated with diabetic complications by association and by meta-analysis studies, and iii) were influenced by disease duration, obesity, number of gestations, glucose serum levels and the use of medications, such as metformin.

**Conclusion:**

This is the first module map study to show the influence of epidemiological, clinical, laboratory, immunopathogenic and treatment features on the transcription profiles of T1D, T2D and GDM patients.

## Background

Diabetes mellitus is considered by the World Health Organization to be a global epidemic. Although the different types of diabetes have varying incidence rates, it is estimated that approximately 6.4% of the world adult population have some type of diabetes [1]. Diabetes is characterized as hyperglycemia resulting from a relative or absolute impairment of insulin secretion as well as peripheral resistance to insulin [2]. Based on etiology, diabetes mellitus has been classified into type 1 diabetes (T1D), type 2 diabetes (T2D), gestational diabetes mellitus (GDM), as well as other types of diabetes, including genetic defects in β-cell function, genetic defects in insulin action, diseases of the exocrine pancreas, endocrinopathies, drug- or chemical-induced forms, infection-induced diabetes, uncommon forms of immune-mediated diabetes, and other genetic syndromes associated with diabetes [2]. The pathogenic mechanisms of each diabetes type are still unclear, especially in the case of T2D and GDM. Pregnant women with gestational diabetes also have an increased risk of developing T2D, suggesting a close relationship between these types [3,4].

T1D is the most studied type of diabetes, has several susceptibility *loci* identified in mice (*Idd1-Idd26*) and is characterized by the autoimmune destruction of pancreatic beta cells leading to insulin deficiency [5]. Macrophages, dendritic cells and lymphocytes are involved in this pathogenic process through a complex interplay of mechanisms implicated in the loss of immune tolerance to autoantigens, including i) the hypoexpression of insulin in the thymus during promiscuous antigen expression [5,6]; ii) autoantigen presentation mediated by molecules coded by the HLA-DRB1*04–DQB1:03:02 and HLA-DR31*03–DQB1*02:01 haplotypes resulting in the development of insulin autoantibodies (IAA) and autoantibodies against the 65 kDa isoform of glutamic acid decarboxylase (GADA), respectively [7]; iii) a deregulation of the immune response mediated by either an impaired expression of surface regulatory molecules (*IL2RA*, *IL2RB*, and *CTLA-4*) or a deregulation of intracellular signals (*PTPN2* and *PTPN22*) [8]; iv) a decreased number of suppressive or T regulatory cells [9]; v) a decreased number of iNKT cells [10]; and vi) a loss of function of molecules involved in the innate immune response [11,12]. In addition, several other genes have been implicated in the development of T1D by human genome wide association studies (GWAS) [13-15], including genes identified by transcriptome analyses in human or animal models [16,17] that evaluated peripheral blood mononuclear cells [18,19], pancreatic beta cells [20] and whole blood cells [21].

In recent years, genetic mechanisms have also been shown to affect the development of T2D [22]. Several studies have associated polymorphisms in the *PPARG* and *KCNJ11* genes with T2D susceptibility [23]. Other studies have identified a strong effect of *TCF7L2* gene variants on T2D risk, possibly affecting proglucagon expression with consequent reduced insulin secretion [23,24]. Indeed, both GWAS and international collaborative efforts to analyze GWAS data from multiple groups, such as the Meta-Analysis of Glucose and Insulin-related traits Consortium (MAGIC), have identified other genetic variants associated with T2D gene susceptibility [25,26], several of which were associated with glycemic traits. Many of these groups of genes were related to abnormal insulin processing (*MADD*)*,* higher proinsulin and lower insulin secretion (*TCF7L2*, *SLC30A8*, *GIPR*, and *C2CD4B*), and abnormalities in early insulin secretion (*MTNR1B*, *FADS1*, *DGKB*, and *GCK*) [22]. Although GWAS have identified susceptibility regions across the genome and transcriptome studies have indicated several modulated genes in beta cells and the usefulness of blood RNA profiles [27,28], functional studies are still needed to understand the role of several genes obtained from association studies.

The pathogenesis of T1D has been considered to be different from that of T2D, and information obtained by GWAS has indicated that most T1D and T2D genetic loci seem to not overlap. However, there is evidence that inflammatory processes involving interleukin-1 may play a role in islet beta cell loss in both types of diabetes [29]. Peripheral blood mononuclear cell (PBMC) energy metabolism has been found to play a major role in the pathogenesis of insulin resistance [30]. In addition, there is increasing evidence that metabolic regulation in these cells could influence the number of PBMCs, proliferation pathways, molecular basal synthesis and leukocyte function [31]. In accordance with these findings, transcriptome studies comparing T1D with T2D [32] and T1D with a control group [18] have revealed changes in the expression of several genes related to inflammatory response, fatty acid biosynthesis, hydrolase activity, detoxification of aldehydes generated by alcohol metabolism and lipid peroxidation, all of which could affect the metabolism of PBMCs in diabetic patients.

Compared with T1D and T2D, GDM has been subject to fewer linkage studies and transcriptome analyses [33]. Many genes, however, are known to be associated with both GDM and T2D, especially those related to obesity and oxidative stress [34,35]. The mechanisms linking excess adiposity to an elevated risk of GDM are not completely understood, but recent evidence points to the role of specific hormones and cytokines known as adipokines, which are secreted by the adipose tissue [36,37]. In addition, transcriptome signatures obtained from placenta [38] and whole blood cells [39] have identified genes involved with lipid metabolism that are differentially expressed between T1D and GDM. Recently, in a meta-analysis of the transcription profiles of T1D, T2D and GDM patients, our group reported that gene expression signatures of GDM patients were closer to those of T1D patients than to T2D [40]. The analyses of gene expression signatures, however, were impaired by the presence of multiple variables associated with each type of diabetes. To circumvent these problems, here we used several bioinformatics tools to analyze demographical, clinical, laboratory, pathogenetic and treatment data against modulated genes that have been annotated by databases containing information regarding both GWAS and gene expression signatures displayed by immune cells. This type of comparison has yielded multiple informative modules that have been used to perform comprehensive maps in cancer [41], and here we apply these tools to diabetes.

## Results

**Figure 1:**
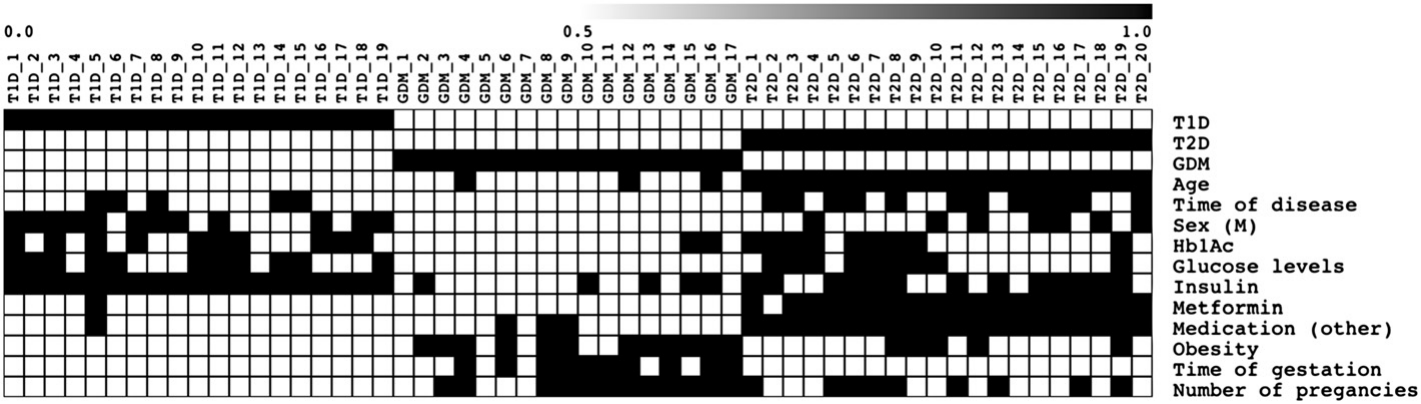
**Heatmap representative of type of diabetes, demographic, clinical, laboratory and treatment features of the patients.** Qualitative variables were assigned by the absence or presence of the characteristic, and quantitative variables were assigned by values below or above the mean values. This information was used as array (experimental) set for the module map construction.

**Figure 2:**
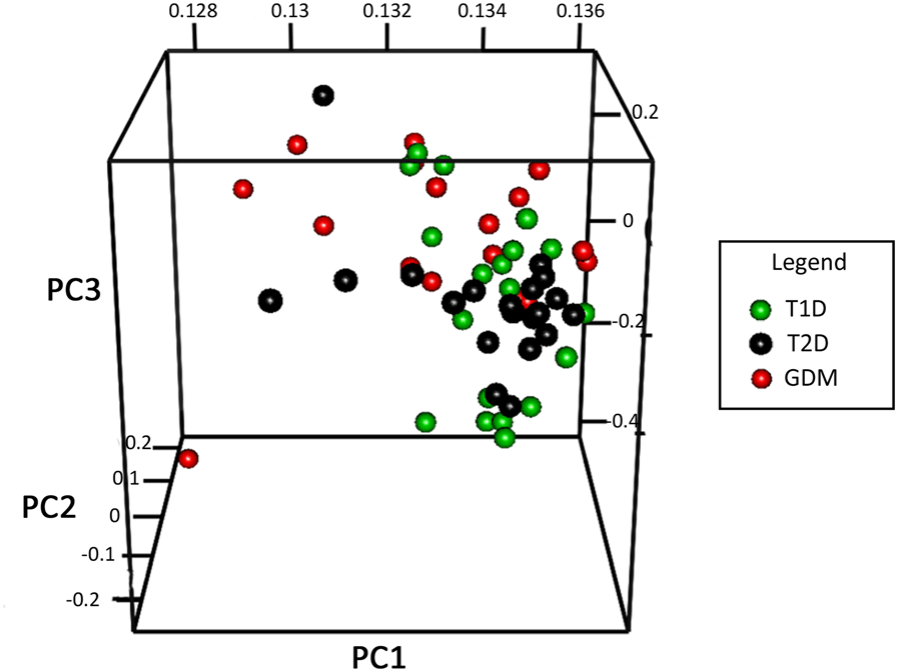
**Principal component analysis (PCA) of the major types of diabetic patients, using the 8,469 informative genes obtained by the DBF-MCL algorithm.** The separation of samples of each type of diabetes after filtering non-informative genes showed similarities among them, indicating that sample transcription profiles were not influenced by the batch effect.

**Figure 3:**
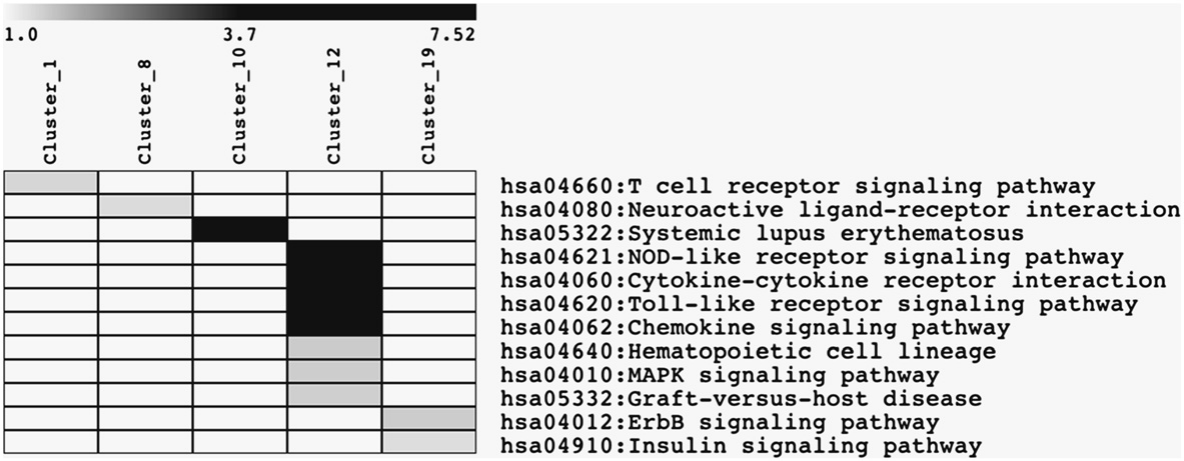
**Heatmap of the significant functional categories of the genes of the clusters obtained by non-informative filters, in the basis of a FDR ≤ 0.1.** The Kegg categories were obtained from DAVID knowledge base with an enrichment *P* value ≤ 0.05 after Benjamini correction. The grey scale represents the logarithm of the enriched *P* value.

Overall, a schematic heatmap with all demographic, clinical and laboratory patient features is shown in Figure 1. The global partitioning analysis of the three types of diabetes disclosed 8,469 transcripts considered as informative, which are available at http://www.rge.fmrp.usp.br/passos/DBF-MCL, the principal component analysis (PCA) of these genes are shown in Figure 2 and the summarized DAVID functional categories (Kegg pathways) of the main clusters are shown in Figure 3.

**Figure 4:**
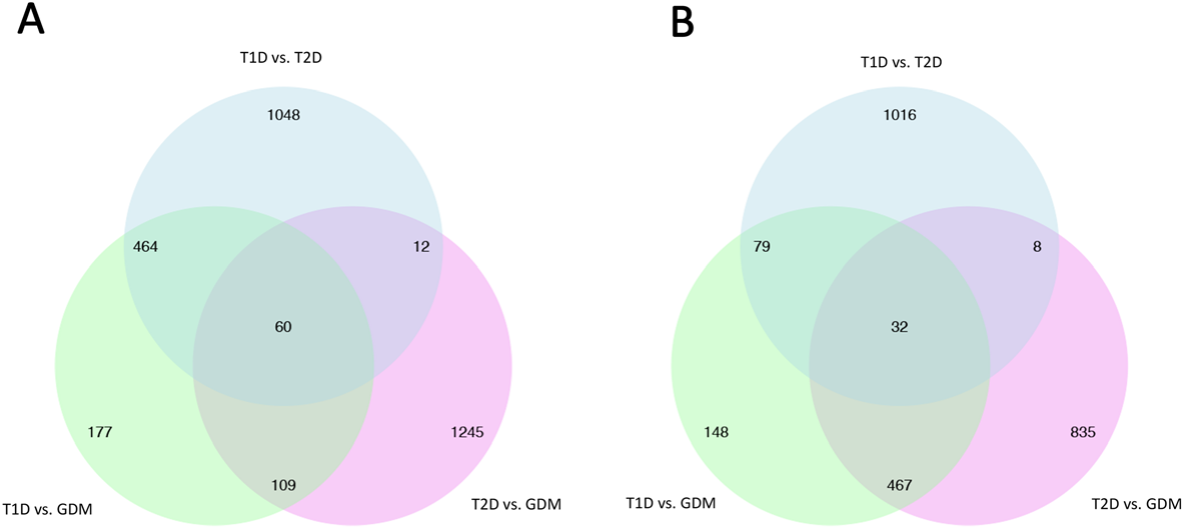
**Venn diagrams show the differentially expressed genes after paired analysis of the three types of DM.** The genes were identified by Rank Product analysis with *P* value ≤ 0.001 and a percentage of false prediction (pfp) ≤ 0.05. The analysis referring to upregulated genes is shown in panel **A** and that of downregulated genes in panel **B**.

**Figure 5:**
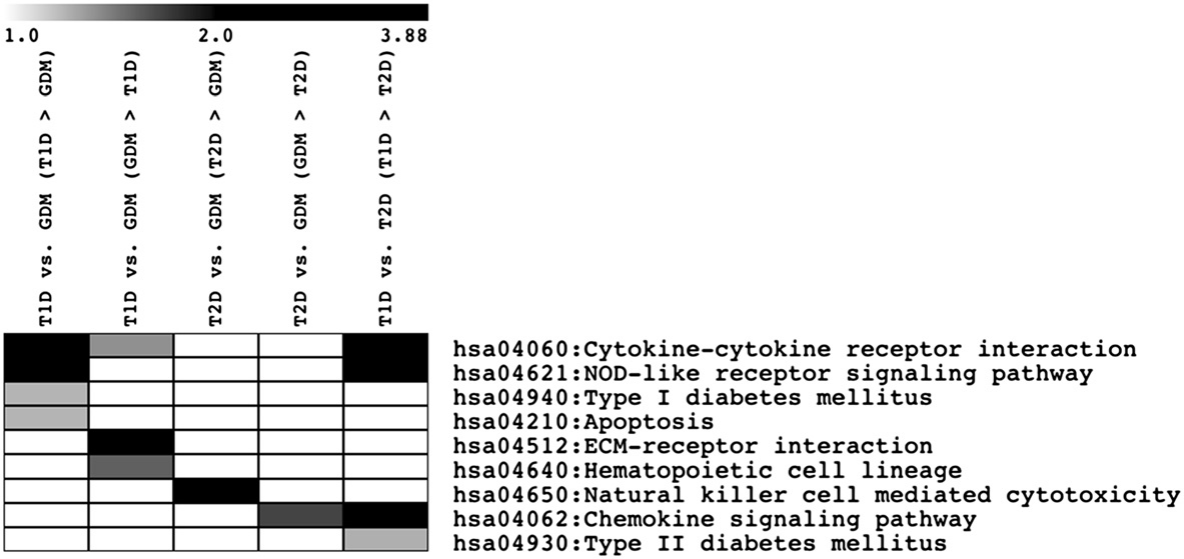
**Heatmap of the significative functional categories of the differentially expressed genes obtained by paired Rank Products analysis with *P *value ≤ 0.001 and percentage of false prediction (pfp) ≤ 0.05 (T1D vs. GDM; T2D vs. GDM and T1D vs. T2D).** The Kegg categories were obtained from DAVID knowledge base with an enrichment pvalue ≤ 0.05 after Benjamini correction. The grey scale represents the logarithm of the enriched *P* value.

**Figure 6:**
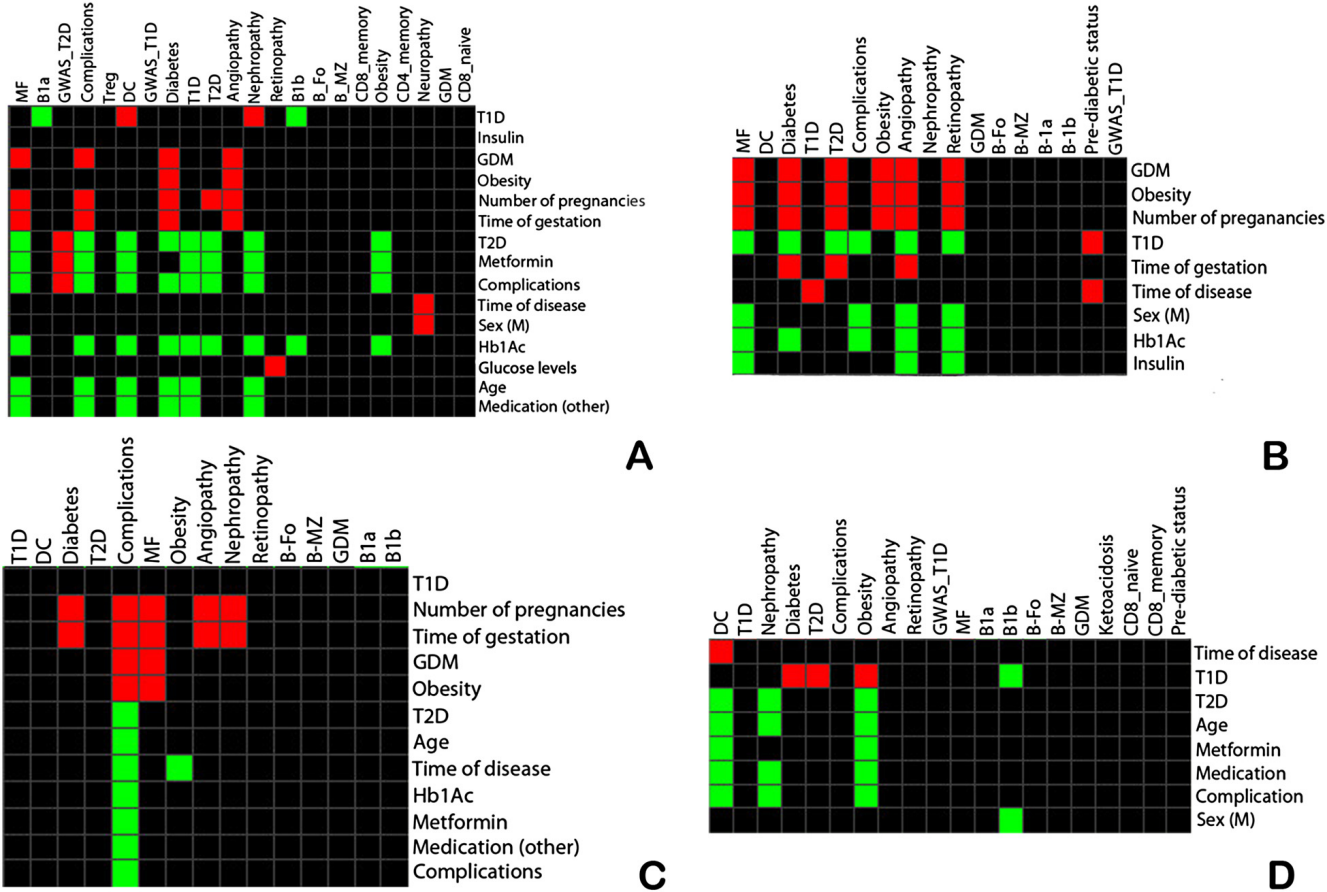
**Heatmaps of the modules identified by Genomica tool, which compares gene lists of immune cells and diabetic association genes with demographic, clinical, laboratory and treatment features of patients (*P *value ≤ 0.05, corrected by the false discovery rate - FDR ≤ 0.05.** The four module maps presented list of genes (induced or repressed), identified by: **A)** Non-informative filters (DBF-MCL algorithm) in the basis of FDR ≤ 10%; **B)** Rank products analysis of T1D vs. GDM; **C)** Rank products analysis of T2D vs. GDM; **D)** Rank Products analysis of T1D vs. T2D. Abbreviations: MF - macrophages ; B1a and B1b - subsets of B lymphocytes; BFo - follicular B lymphocytes; BMz - marginal zone B lymphocytes; Treg - regulatory T lymphocytes; DC - dendritic cells ; CD4 and CD8 - subsets of T lymphocytes.

**Figure 7:**
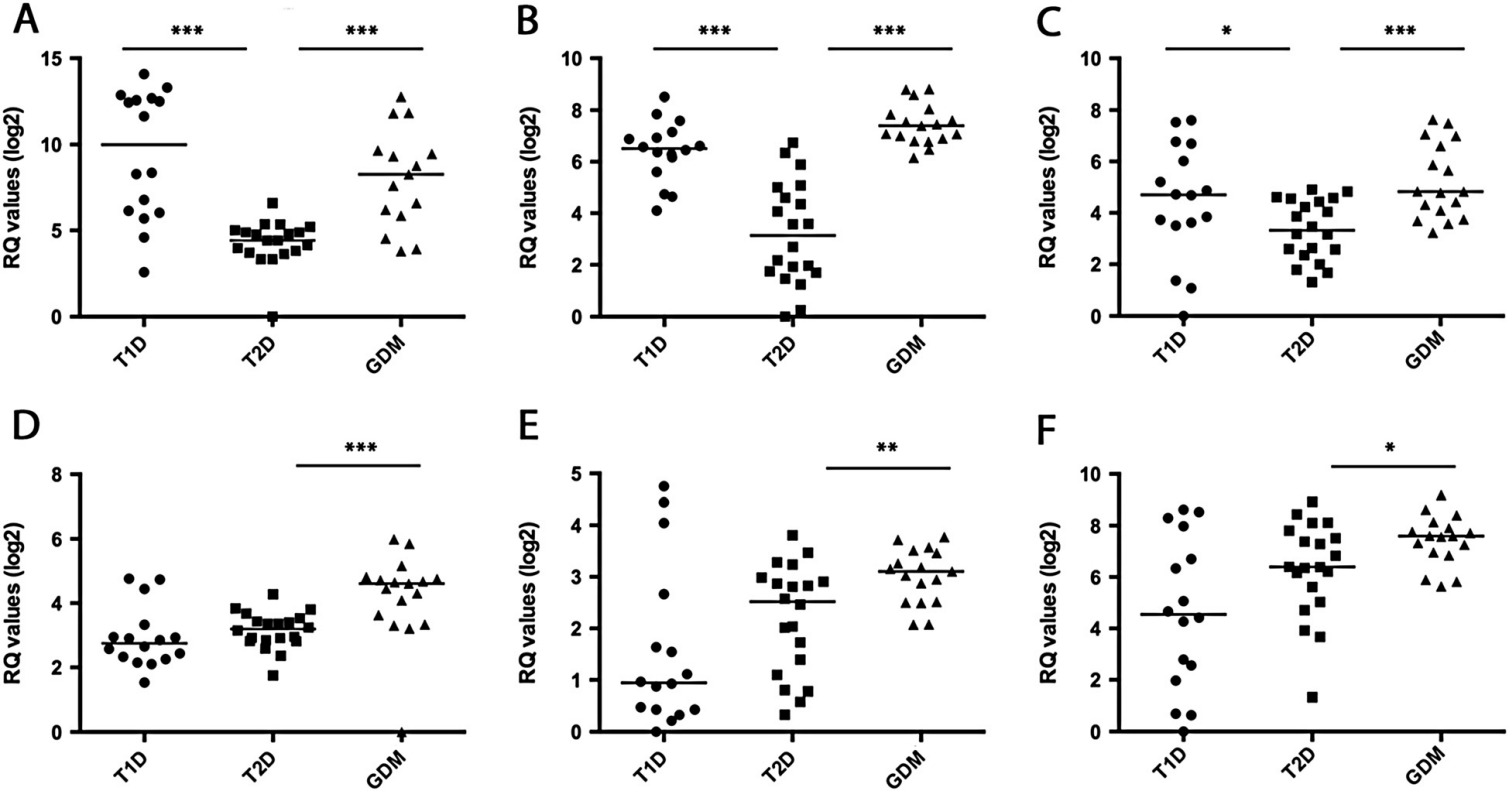
**Confirmation of microarray findings by qRT-PCR of (A) *IL1B, *(B) *RGS1, *(C) *EGR2, *(D) *FOXO3A, *(E) *SOD2 *and (F) *HIF1A *genes.** Expression levels were normalized to *HPRT1*. The differences were evaluated by Mann–Whitney U test. * p < 0.05; ** p < 0.01 and *** p < 0.001 were considered significant.

The Venn diagrams yielded shared and specific genes after statistical analysis by rank products (T1D versus T2D, T2D versus GDM and T1D versus GDM) (Figure 4) as well as multiple significant summarized DAVID functional categories (Kegg pathways) (Figure 5). The module maps encompassing all analyses, i.e., genes obtained from both partitioning and rank products, were created with the set of genes obtained in each of the approaches described above (Figure 6). Finally, the confirmation by PCR analysis of important genes involved in diabetes is shown in Figure 7.

### Global analysis of the data set with density-based filter and Markov clustering (DBF-MCL) and principal component analysis (PCA)

A list of 8,469 genes obtained from global partitioning analysis (DBF-MCL method applying a non-informative filter) yielded informative non-supervised clusters of co-regulated genes. Figures showing all non-supervised clusters and the respective gene list are available at http://www.rge.fmrp.usp.br/passos/DBF-MCL. A PCA plot of filtered genes is shown (Figure 2). Despite we have more similarity between T1D and GDM, some patients (with the lowest levels of glycemic levels) were closer to the other types. Overall, many gene expression profiles are shared between T1D and GDM. A typical example of this analysis can be observed in cluster #1 (3,089 probes), in which all T2D patients shared a group of induced genes encoding zinc finger proteins, while these same genes were found repressed in T1D and GDM patients. Similarly, in cluster #12 (216 probes), all T2D patients shared a group of repressed genes associated with cytokine and chemokine activity, NOD-like and Toll-like receptor signaling pathways and MAPK signaling pathway (Figure 3).

### Supervised analysis of T1D, T2D and GDM patients

To understand the influence of demographic, clinical, pathogenetic and laboratory features on the differential gene expression profiles among diabetes patients, a second strategy was used. Module maps were created by comparing the three groups at the same time (from the partitioning analysis) as well as by the individual two by two group comparisons. These paired analysis module maps were constructed based on the results obtained from statistical analyses of rank products and are represented in Venn diagrams (Figure 4), disclosing up and down-regulated genes shared by the same type of diabetes in different analysis. The functional categories of these genes are shown in Figure 5.

T1D patients presented several genes that were induced when compared to GDM patients, including genes in the MHC region (*HLA-DQA1* and *HLA-DQA2*), TNF receptors (*TNFRSF17*, *TNFAIP6*), cytokines (*IL1A*, *IL-1B*, *IL1F10*, *IL4*, *IL6*, *IL8*, *IL23A*, *IL27*) and cytokine receptors (*IL1R2*, *IL1R1N*, *IL18R1*), chemokines (*CXCL1*, *CXCL2*, *CCL20*, *CCL23*, *CCL3L3*, *CCL4*) and chemokine receptors (*CCR3*, *CRL2*), lymphocyte receptors (*KIR3DL2*, *KIR2DS4*), and transcription factors (*GLIS2*, *SOX8*, *GATA2*, *RUNX1*, *SOD2*, *FOXC1*, *FOXC2*, *FOXE1*). Additionally, T1D patients presented several genes that were differentially expressed compared to T2D patients, including immune response genes, as MHC region genes (*HLA-DQA1* and *DQA2*), clusters of differentiation genes (*CD8B, CD55, CD83*), cytokines (*IL1A*, *IL1B,IL6, IL8, IL23A*), chemokines (*CXCL1*, *CCL3L3, CXCL2, CXCL3, CCL20, CCL23, CCL24, CCL3, CCL4*), NOD-like receptor signaling pathway (*CXCL1, IL6, CARD9, TNF, IL8, CXCL2, CASP8, NFKBIA, IL1B, MAPK8, TNFAIP3, NLRP3*) and transcription factors (*GLIS2, SOX8, FOXC2, FOXC1, FOXE1, FOXK1*).

GDM patients presented several genes that were induced compared to T1D patients, including toll-like receptors (*TLR6*, *TLR7*), carbohydrate binding genes (*LGALS3*, *LGALS12*, *CLEC7A*, *CLEC1B*), plasma membrane receptors (*IL5RA*, *CCR1*), MHC genes (*HLA-DRB3*), solute carrier family genes (*SLC6A10P*, *SLC6A4*, *SLC1A5*, *SLC4A1*, *SLC8A1*, *SLC6A17*, *SLC16A3*, *SLC6A8*, *SLC14A1*), cluster of differentiation genes (*CD9*, *CD33*, *CD36*), and chemokines (*CXCL5*, *CCL15*, *CXCL12*, *CXCL10*). In contrast, GDM patients presented several genes that were induced compared to T2D, including immune response genes (*CXCL10*, *CXCR4*, *CD46*, *CCL3L3, IL1B, IL1A, NFKBIZ, IL27, C1QA, CD83, C1QB, HIF1A, DEFA4, DEFA3, KIR2DL4, CXCL1, CCL3, TNF, CCR1, CXCL3, CXCL2, CCL4, CCL23, CCL20, IL6, CR2, IL8, IL1RN STAT3, CD55*) and genes involved in the response to hormone stimulus (*PTGS2, LDLR, PGF, PDGFA, PTGS1, NOS3, ADAM9, EGR2, SOCS2, SOCS3, RXRA, ADIPOR1, GAL, JUNB, C1QB, RETN, ADM, SORT1, CAV2, CAV1, TNF, GRB2, ERBB3, GNG11, BCL2L1, PIK3R1, IL6, IL1RN, STAT1, STAT3, CDKN1A, BMP7, IGFBP2*).

T2D patients presented several genes that were differentially expressed compared to T1D patients, including genes of numerous zinc finger proteins and genes regulating transcription (*ZNF184, ZNF576, ZNF449, ZNF594, ZNF641, ZNF100, ZNF644, ZNF189, ZNF785, ZNF436, ZNF613*, among others). Finally, the comparison between T2D and GDM showed multiple genes induced in T2D patients, including genes involved in DNA-binding, as zinc finger (*ZNF582, ZNF250, ZNF184, ZNF181, ZNF775, ZNF773, ZNF248, ZNF397, ZNF578, ZNF442, ZNF443,* among others).

### Comprehensive functional analysis using a module map approach

To identify the influence of patient features (array or experimental sets) on gene information (gene sets), we constructed several module maps, stratifying patients according to demographic, clinical, laboratory and therapeutic characteristics. We used specialized databases associated with diabetes complications [42], gene clusters associated with diabetes obtained from association studies (GWAS) [43], and isolated immune cell types associated with the pathogenesis of diabetes [44]. The most relevant modules are shown in Figure 6.GDM patients exhibited up-regulated genes observed in diabetes complications (including angiopathy) and in macrophages. GDM, number of gestations per patient and gestation time were associated with the induction of diabetic complications genes. Interestingly, a history of 2 or more gestations was positively associated with the modulation of T2D genes, while in T1D patients the number of gestations did no influence the transcription profile of female patients (Phenopedia). T1D patients exhibited induced genes typical of those displayed by dendritic cells and repressed genes typical of those presented by B-lymphocytes. In addition, T1D patients exhibited induction of genes related to diabetes nephropathy. The use of insulin did not influence gene expression patterns; however, increased serum glucose level was associated with the induction of genes related to diabetic retinopathy. In patients with T2D, the disease itself as well as the use of metformin was associated with the repression of genes implicated in obesity and diabetic complications. In contrast, genes found by T2D GWAS were associated with genes induced in T2D patients and the use of metformin. Male patients who had T2D for 11 or more years were at increased risk of neuropathy. The heatmap shown in Figure 6A illustrates these results.In the paired analysis of T1D and GDM patients, both obesity and a history of two or more gestations were positively associated with T2D development. Conversely, in T1D patients, these variables were negatively associated. Additionally, in T1D patients, the use of insulin repressed genes of angiopathy and retinopathy, and macrophage gene expression was associated with GDM (Figure 6B). In the paired analysis of T2D and GDM, the number of gestations was positively associated with genes related to diabetic complications. The use of metformin was negatively associated with complications, and these same genes were repressed in T2D (Figure 6C). The paired analysis of T1D and T2D patients showed repression of dendritic cell genes associated with T2D and the induction of dendritic cell genes associated with time of disease. Obesity genes were also induced in T1D patients (Figure 6D).

### Confirmation by real-time PCR

We performed the confirmation of *EGR2*, *RGS1*, *FOXO3A, HIF1A, IL1B* and *SOD2* genes (Figure 7)*,* which were chosen because some of these genes have been previously described as specific for GDM compared with T2D (*SOD2, FOXO3A, HIF1A*). Compared to GDM, these genes were downregulated in T2D as evaluated by microarrays and qRT-PCR, presenting fold changes of 4.34, 1.75 and 1.72, respectively (microarrays), and 1.51, 2.66 and 2.31, respectively (qRT-PCR). In addition, compared to T2D, the *EGR2, IL1B and RGS1* genes were downregulated when compared to T1D (fold changes of 1.82, 13.96 and 3.08, respectively, for microarrays and 2.59, 47.83, and 9.95, respectively, for qRT-PCR), and downregulated when compared to GDM (fold changes of 2.22, 11.11 and 4.34, respectively, for microarrays, and 2.82, 14.50, and 18.36, respectively, for qRT-PCR).

## Discussion

Diabetes mellitus is one of the most studied diseases, and a large amount of information is available in the public databases regarding genetic association, meta-analysis and associated complications. Few studies, however, have systematically compared the major types of diabetes in terms of gene expression profiles at the genomic level. In this study, we performed an integrative analysis of the transcriptome profiles of the major types of diabetes using several bioinformatics tools. The major innovation of this study was the construction of informative module maps integrating epidemiologic, clinical, laboratory, pathogenetic, genetic, and molecular factors implicated in diabetes to identify individual and shared features in T1D, T2D and GDM.

The major finding of our global partitioning analysis that examined the differences in transcription among each diabetes patient was the cluster of genes associated with inflammation. The high expression levels of these genes in some T1D and GDM patients seem to influence the global gene expression pattern of diabetic patients. Indeed, several important molecular mechanisms identified by clustering account for an intricate array of inflammation pathways. One important finding was the up-regulation of many mediators of the NOD-like receptor signaling pathway. It has recently been suggested that NOD-like receptors can be induced by hyperglycemia and oxidative stress products, which could link metabolism and inflammation, particularly through the participation of *IL1B*[45]. In this study, many genes involved in these pathways were induced in T1D and GDM patients, including *NLRP3* (an important receptor of the NOD-like pathway), *IL1B, CXCL1, CXCL2, IL6, IL8, TNF, RIPK2, TNFAIP3* and *NFKB1A. TNF* and *IL1B* are strongly involved in the regulation of nitric oxide biosynthetic processes [46] and are regulated by SOD2 [47]. In addition, other upregulated genes including chemokines (*CCL3, CCL4*), cytokines (*IL6, IL8, TNF*) and transcription factors (*NFKBIA, MAPK8*) are involved with other inflammatory processes, such as toll-like receptors signaling pathway, and the expression of *IL1B* and *SOD2* genes was confirmed by real-time PCR. Curiously, genes associated with these processes were downregulated in T2D compared with the other types of diabetes. To understand this result, we took advantage of the rank product analysis (paired analyses), which showed particular features of the involvement of these inflammatory pathways in each type of diabetes. The comparisons between T2D and GDM as well as between T1D and T2D revealed several interesting results. At the same time that NOD-like receptors were induced in T1D and GDM, hundreds of zinc finger protein genes were induced in T2D. Additionally, drug treatment with metformin in T2D patients seems to influence the gene expression patterns, whereas insulin treatment did not. As literature findings have indicated that the expression of transcription factors associated with T2D can be induced by hypoglycemiants, it is possible to hypothesize that these drugs may induce alterations in the normal T2D expression profile. Finally, the comparisons between T2D and T1D reveal the repression of important genes associated with T2D diabetes, particularly transcription factors involved in glucose homeostasis (*TCF7L2*) [24,48], NAD + (*FOXO3*) [49] and regulation of cellular and systemic response to hypoxia (*HIF1A*) [50], and the differential expression of *HIF1A* and *FOXO3A* in our series was also confirmed by real-time PCR.

To further understand the close similarity between the transcription profiles in T1D and GDM, we examined the paired comparisons between these types. T1D patients exhibited modulation of genes in the MHC region, including the induction of *HLA-DQA1* and *HLA-DQA2*. The *DQA* gene encodes the alpha chain of the HLA-DQ heterodimeric molecule. HLA-DQ is non-covalently associated with DQβ, which has been associated with T1D susceptibility in multiple populations [51-54]. In contrast, few studies have associated any HLA class II genes, including *HLA-DRB3* genes, induced in GDM [53]. Although a role of HLA class II genes in T1D pathogenesis has not yet been established, decreased surface expression of HLA-DQ molecules on CD4 and CD8 peripheral cells has been reported in recently diagnosed T1D patients exhibiting *DQB1* susceptibility alleles. This finding was attributed to the instability of these molecules on the cell surface [54]. As none of our patients were recently diagnosed, it is interesting to observe that the expression of *DQA* MHC molecules is still modulated even after the disease has been present for long periods of time. On the contrary, there is also evidence that humoral responses to autoantigens may be driven by the *HLA-DQA1* genes even in recently diagnosed patients [55]. MHC susceptibility alleles associated with T1D (DQA1*05:01, DQB1*02:01/DQB1*03:02) are different from those associated with GDM [56]. In addition to MHC genes, T1D patients also showed different levels of the killer immunoglobulin-like receptor (*KIR3DL2* and *KIR2DS4*) family genes compared with GDM patients (*KIR2DL4*). There are no gene expression studies implicating KIRs in diabetes. These findings suggest that the close similarity of the T1D and GDM transcription profiles may be due to the overall inflammatory gene patterns observed in both conditions, including the modulation of several genes primarily involved with the innate immune response.

Previously, the construction of module maps for cancer patients has revealed important modules that characterize different cancer lineages [41], allowing the identification of cancer biomarkers. Major findings regarding each group of diabetes patients are discussed below, including epidemiological, clinical, laboratory, genetic and pathogenetic features. In all module maps in which the gene profile of GDM was compared with other types of diabetes, we observed an up-regulation of genes typically expressed by macrophages. Overall, the comparison with genesets with arraysets yielded positive associations, except for T1D, which may be attributed to differences in patient age and the autoimmune nature of T1D. These macrophage genes were co-regulated with those appearing in diabetic complications such as angiopathy and retinopathy, including the *IL1B* and *RGS1* genes, as seen in Figure 6A, B and C. As we also observed that increased glucose levels were associated with the development of retinopathy, it is possible that the effect of high glucose levels on macrophages might be involved in the creation of GDM complications. Moreover, GDM patients exhibited gene profiles similar to those reported for obesity (data available in the Phenopedia public data banks), and the majority of patients in our study were overweight. It is also interesting to observe that obesity, two or more gestations per patient, and gestations lasting over 30 weeks exhibited the same modules of induced genes in GDM patients, as observed in Figure 6A. Additionally, in GDM patients, having two or more gestations was positively associated with the development of T2D. Indeed, it is important to note that obese GDM patients have an increased risk of developing T2D [57]. This information is important for future studies of GDM.

In T1D patients, we observed a positive association with the profile reported for dendritic cells (ImmGen) (Figure 6A), particularly in patients exhibiting long-term disease (Figure 6D). As dendritic cells play an important role in antigen presentation via MHC class II molecules and as MHC class II genes are also induced in long-term T1D patients, it is possible to hypothesize that abnormal antigen presentation (foreign or self) is a chronic phenomenon in T1D. In contrast, T1D patients also exhibited a repression of genes associated with B1a and B1b lymphocytes (naïve B cells), in which the B1a lymphocyte subtype (CD5+) has been associated with the production of natural and autoantibodies [58]. Unfortunately, until the moment, there is no public data regarding the gene pattern of activated B cells. Another important finding is the similarity of T1D gene expression profiles with that observed for diabetes nephropathy (Phenopedia). As no patients in our analysis exhibited clinical nephropathy, it may be valuable to further study the particular patients exhibiting nephropathy related gene patterns.

Finally, T2D patients exhibited gene expression profiles that were in disagreement with those reported in the public databases (Phenopedia) for diabetic complications (Figure 6C). Considering that half of our T2D patients exhibited higher median glucose levels than our T1D and GDM patients, one could expect to find the induction of genes associated with diabetic complications. As the T2D patients were treated with many types of medications besides hypoglycemiants, it is possible that this intriguing and unforeseen finding may be a consequence of these treatments. Another possible explanation may be related to inflammation genes, which were down-regulated in T2D compared to T1D and GDM patients. The coupling of inflammation and drugs used to treat T2D might be responsible based on these findings. Indeed, the use of metformin and other medications (aspirin, captopril, atorvastatin, and hydrochlorothiazide) seems to modulate the expression of a large number of genes (Figure 6C and D), possibly affecting the inflammation status of T2D patients. Some of the down-regulated genes observed in T2D patients being treated with several drugs included *IL1B, IL4, IL8, CCL2* and *TNF*. All of these genes are involved in inflammation and are also modulated in macrophages (data not shown). However, we cannot disregard the participation of the inflammatory pathway in T2D, as genes that participate in the NOD-like receptors signaling pathway probably also play a role in T2D [45]. Recent findings have shown that the down-regulation of *FOXO1* expression in macrophages blocks lipid accumulation in these cells, affecting many processes [59]. In our analysis, *FOXO3A* was also down-regulated in T2D patients compared to GDM. Both *FOXO1A* and *FOXO3A* genes are mediators of the same signaling pathway, AMPK, which can be activated as a consequence of long-term metformin use [49], and the expression of *FOXO3A* was confirmed by real-time PCR.

In conclusion, our analysis revealed that T1D and GDM exhibited a similar up-regulation of inflammatory genes.

## Methods

### Study population

We studied 56 adult diabetic patients, 19 presenting T1D (7 women/12 men) with ages of 18–36 years, 20 presenting T2D (13 women/7 men) with ages of 41–72 years and 17 presenting gestational diabetes with ages of 23 to 40 years. For our analysis, we used the mean age of the three groups (37 years with SD ± 14). The mean length of disease was 11 ± 5.3 years, while the mean values of glucose and Hb1Ac levels were 130.7 mg/dL with SD ± 75.4 and 8.9 with SD ± 1.8, respectively. For gestational diabetes patients, the mean period of gestation was 30.5 with SD ± 5.7 weeks, and the mean number of pregnancies was 2 ± 1 per patient. Twelve GDM women had more than two gestations, while 9 T2D and none T1D women presented this characteristic. T1D and GDM patients were treated only with insulin, while T2D patients were treated with insulin in combination with metformin, captopril, aspirin, atorvastatin and hydrochlorothiazide. All patients underwent follow–up examinations at the Outpatient Clinics of the Division of Endocrinology, Faculty of Medicine of Ribeirão Preto, University of Sao Paulo, Brazil. The exclusion criteria were based on recent episodes of ketoacidosis, active nephropathy, proliferative retinopathy, diabetic foot syndrome, high LDL levels and diagnosed cardiovascular diseases. Figure 1 shows a schematic heatmap with all demographic, clinical and laboratory patient features. The study protocol was approved by the local Ethics Committee (Comitê de Ética em Pesquisa do Hospital das Clínicas e da Faculdade de Medicina de Ribeirão Preto da Universidade de São Paulo, under the permit # 9153/2008), and informed consent was obtained from all participants.

### Blood collection, peripheral mononuclear cell isolation and RNA extraction

A total of 20 mL of peripheral blood was collected and used for the isolation of PBMCs by discontinuous gradient density centrifugation on a Ficoll-Hypaque cushion (Sigma, St. Louis, MO). Total RNA was extracted using the Trizol reagent (Invitrogen, Carlsbad, CA) according to the manufacturer’s instructions. RNA concentrations and ratios were checked using a NanoDrop ND-1000 spectrophotometer (NanoDrop Products, Wilmington, DE), and the RNA integrity was assessed by microfluidic electrophoresis using a 2100 Bioanalyzer and RNA 6000 nanochips (Agilent Technologies, Santa Clara, CA). We used only samples that exhibited median RNA integrity number (RIN) ≥ 9.0.

### Oligo microarrays

Hybridizations onto whole human genome 4x44K oligo microarrays (G4112F, Agilent) were performed using the one color (Cy3) Quick Amp labeling kit (Agilent). Briefly, 500 ng of total PBMC RNA plus spike-in controls were reverse transcribed into double stranded cDNA. The primers used for this reaction contained many consecutive thymine bases attached to a T7 promoter that paired at the 5’ end of the first strand of cDNAs. Next, the T7 polymerase was added along with nucleotides labeled with fluorescent cyanine-3 (Cy3) dye, which amplified the anti-sense complementary RNAs (cRNA). The cRNAs were purified and then hybridized to the microarray for 17 hours at 65°C. After washing, the slides were scanned using a DNA Microarray Scanner with Surescan High-Resolution Technology (Agilent). A complete file providing microarray data from all samples used in this study, as well as the numerical quantitative data and experimental conditions, is available on line at the ArrayExpress public database [60] through the following accession numbers: T1D (E-MEXP-3348), T2D (E-MEXP-3287) and GDM (E-MEXP-3349). These data correspond to a part of our laboratory databank, which were made publicly available and can be used for further studies and/or reanalysis.

### Data quantification and normalization

Data quantification and quality control were analyzed using the Feature Extraction (FE) software version 10.7 (Agilent Technologies). Expression data were loaded into an R-environment [61] using the AgiND package [62], a tool developed by the Technologies Avancées pour le Genome et la Clinique (INSERM U1090, Marseille, France). The AgiND tool is available by request [63]. The background adjustment was performed by subtracting median background values from the median expression values obtained by FE and then converting the results to log-scale. For each sample, any negative values observed were replaced by randomly selected small positive values. After log-transformation, quantile normalization using the normalizeQuantile function was performed.

### Density based filtering and Markov clustering (DBF-MCL) and principal component analysis (PCA)

The Density Based Filtering and Markov Clustering (DBF-MCL) algorithm implemented in the Rtools4TB bioconductor package [64,65] was used to extract sets of co-regulated genes from our microarray dataset. DBF-MCL is a tree-step adaptive algorithm that finds genomic elements and genes located in dense areas, uses selected items to construct a graph and finally creates a partitioning graph using the Markov Clustering Algorithm (MCL). A 10% false discovery rate (FDR) was used. The principal component analysis (PCA) was performed from the informative genes identified by DBF-MCL. In this analysis, the R function prcomp [66] was used to evaluate PCA and the rgl package [67] to construct 3D graphics.

### Statistical analysis

Differentially expressed genes were identified using the R package RankProd [68,69]. Although the non-parametric rank product method does not make any assumptions about the data distribution, it can provide frequency ranking scores at each data point and is thus a robust tool for creating ranking lists. Genes were considered significantly expressed when they presented p-values and percentage of false positive predictions (PFP) smaller than 0.001 and 0.05, respectively. This test was used to perform paired analysis between T1D *versus* GDM, T2D *versus* GDM and T1D *versus* T2D. Venn diagrams illustrating up and down-regulated genes in each analysis are shown in Figure 4.

### Module-map construction

Significant and differentially expressed genes obtained by the use of DBF-MCL and Rank Products were initially clustered using the Cluster 3.0 and TreeView softwares [70,71]. Then, module maps were constructed using an ensemble of tools provided by the Genomica software [41], which searches for higher-order modules of gene sets and samples. Initially, this algorithm uses gene sets to annotate genes that were up-regulated (or down-regulated) by at least 1.5-fold. The algorithm compares the modulated genes with array sets, including groups of compartmentalized genes, and organizes them into modules (module maps) discriminating variable-specific gene patterns according to patient features. It was used *P* value ≤ 0.05 with false discovery ration (FDR) ≤ 0.05.

### Module map array set variables

The variables used to create the experimental sets included demographic parameters (age and gender), clinical variables (disease duration, obesity, duration and number of gestations), laboratory data (serum glucose and glycated hemoglobin levels), and treatment features (use of hypoglycemiants such as insulin, metformin or other drugs). All input variables were transformed into binary data (0 or 1), according to the nature of the variable, i.e., qualitative variables were assigned by the absence or presence of the characteristic, and quantitative variables were assigned by values below or above the mean values. The variables used included age, time of disease, gender, serum glucose and glycated hemoglobin values (Hb1Ac), use of insulin, use of metformin, use of any other type of medication, obesity, gestation time (for GDM patients) and number of gestations (for GDM patients) (Figure 1).

### Module map gene set variables

Immune cell specific gene sets were obtained by re-analyzing raw data from the ImmGen project [44,72] (the detailed procedure is provided as Additional file 1). Disease-related gene sets were obtained from GWAS integrator [43] and Phenopedia [42]. GWAS integrator is a compilation of genes offered by GWAS catalogs and databases such as HapMap, SNAP and HugeNavigator [73]. All of the information about genes related to T1D and T2D were considered for use in this study. Phenopedia is an available database about genetic association studies and meta-analysis summarized in the Human Genome Epidemiology (HuGE) encyclopedia [74]. Lists of genes can be obtained about specific diseases or related complications [75]. The available lists of genes related to diabetes mellitus include those associated with pre-diabetic status, diabetes, type 1 diabetes, type 2 diabetes, gestational diabetes, diabetic complications, obesity, angiopathy, ketoacidosis, nephropathy, neuropathy and retinopathy.

### Functional analysis

All clusters of co-regulated genes were analyzed by functional analysis using the Database for Annotation, Visualization, and Integrated Discovery (DAVID) version 6.7 [76]. This approach was used to identify significant biological processes and pathways represented by the differentially expressed genes [77]. A biological process or pathway was considered to be significant if it contained a minimum of three genes per category featuring score values less than 0.05 including the Benjamini-Hochberg correction. Moreover, we selected Kyoto Encyclopedia of Genes and Genomes (KEGG) provided by DAVID analysis [76] to explore biological pathways.

### Confirmation by real-time PCR

Relevant and differentially expressed genes obtained using the microarray data were confirmed using qRT-PCR (Table 1). The sequences of selected genes were retrieved from the NCBI GenBank database [78]. All major alternative transcripts were considered to design the primers, and the Primer3 web tool [79] was used to select pairs of oligonucleotide primers spanning at the exon/exon junction. An optimal melting temperature of 60°C was standardized for all genes. Transcriptional expression levels were determined using SYBR Green (Life Technologies), in a StepOne Real Time PCR System (Applied Biosystems). The experiments were done in duplicates. The *GAPDH* and *HPRT1* genes were used as constitutive genes and the selection of the best genes for qRT analysis was done using the Housekeeper software [80]. The standard curve for each primer was determined and only primers with efficiency upper than 90% were used. It was applied the ΔΔCT method as described by [81], and the minimum expression value was used as calibrator. The graphics were constructed in GraphPad Prism version 6.0 and statistical analysis in the R statistical environment version 3.0.1. Non-parametric Mann–Whitney U test was used, considering *P* value ≤ 0.05 to be significant.

**Table 1:** **Primers used for confirmation by qRT-PCR of the *IL1B, RGS1, EGR2, FOXO3A, SOD2 *** **and *HIF1A *** **genes**

**Gene**	**Accesion numbers**	**Primer forward**	**Primer reverse**	**Size (bp)**
***IL1B***	NM_000576.2	5′-CCACAGACCTTCCAGGAGAA-3′	5′-GTGATCGTACAGGTGCATCG-3′	121
***RGS1***	NM_002922.3	5′-TGGCTGGCTTGTGAAGACTA-3′	5′-GATTCTCGAGTGCGGAAGTC-3′	131
***EGR2***	NM_000399.3	5′-GGTGACCATCTTTCCCAATG-3′	5′-TATGGGAGATCCAACGACCT-3′	120
***FOXO3A***	NM_001455.3	5′-GTGCTAAGCAGGCCTCATCT-3′	5′-TTGGCAAAGGGTTTTCTCTG-3′	119
***SOD2***	NM_001024465.1	5′-GACAAACCTCAGCCCTAACG-3′	5′-TTGGACACCAACAGATGCAG-3′	124
***HIF1A***	NM_181054.2	5′-TCAGCTATTTGCGTGTGAGG-3′	5′-AAAACCATCCAAGGCTTTCA-3′	107
***GAPDH***	NM_002046.3	5′-CTCTGCTCCTCCTGTTCGAC-3′	5′-ACGACCAAATCCGTTGACTC-3′	112
***HPRT1***	NM_000194.2	5′-GACCAGTCAACAGGGGACAT-3′	5′-CTGCATTGTTTTGCCAGTGT-3′	111

*GAPDH* and *HPRT1* were selected as endogens.

## Conclusion

The present study revealed that epidemiological, clinical, laboratory, immunological, genetic and treatment features influenced the transcriptome profiles in the major types of diabetes. In addition, inflammation associated with macrophage and dendritic cell function may be responsible for clustering GDM and T1D patients together, while inflammation in T2D may be influenced by drug treatment.

## Competing interests

The authors declare that they have no competing interests.

## Authors’ contributions

Wrote the paper: AFE GAP DP EAD. Conceived the study and participated in its design and coordination: AFE GAP CN DP EAD. Provided material and analytical tools: ETSH GAP CN DP EAD. Performed patient selection, sample collection and cell separation: AFE DJX DMR. Responsible for diabetic patient’s treatment and clinical information data: MCFF MFF. Performed RNA extraction and sample quality control: AFE DJX. Responsible for hybridization and acquisition of microarray data: AFE DJX CCVA FSMC CM. Performed data analysis: AFE DP. All authors contributed to and approved the final manuscript.

## Pre-publication history

The pre-publication history for this paper can be accessed here:

http://www.biomedcentral.com/1755-8794/7/28/prepub

## Supplementary Material

Additional file 1 ImmGen supplementary data. Click here for file
