# Supplementary material for: Integrative analysis of the transcriptome profiles observed in type 1, type 2 and gestational diabetes mellitus reveals the role of inflammation
Source: BMC Med Genomics. 2014 May 23;7:28. doi: 10.1186/1755-8794-7-28 (PMC4066312; doi:10.1186/1755-8794-7-28)
Supplement: Additional file 1 — ImmGen supplementary data. [file 1755-8794-7-28-S1.doc]

**ImmGen supplementary data**

We analyzed a compendium of mouse immune cell Affymetrix raw microarray data offered by the ImmGen project members, with all the ethics conditions respected. The objective was to generate gene annotations from immune cell types to compare with PBMC population of diabetic patients and create gene sets for module map analysis. We used a threshold of 2-fold of median ratio of all samples and after this procedure we checked if the main markers which characterized each cell subtypes were present. To circumvent problems related to the differences between tissues of isolation, we selected shared genes of all samples. To obtain human signatures from the data, we searched for ortholog genes from Homologene database.

Table 1. Immune cell samples used and the number of genes obtained after analysis.

| **Samples** | **Identification (ImmGen)** | **Immuno-markers** | **Number of genes** |
| --- | --- | --- | --- |
| **B1a.Sp.1** | B-1a (spleen) | CD19+B220+IgM++AA4-CD23-CD43+CD5+ | 176 |
| **B1a.Sp.2** | B-1a (spleen) | CD19+B220+IgM++AA4-CD23-CD43+CD5+ | 181 |
| **B1a.Sp.3** | B-1a (spleen) | CD19+B220+IgM++AA4-CD23-CD43+CD5+ | 192 |
| **B1a.PC.1** | B-1a (peritonial cavity) | CD19+B220+IgM++AA4-CD23-CD43+CD5+ | 206 |
| **B1a.PC.2** | B-1a (peritonial cavity) | CD19+B220+IgM++AA4-CD23-CD43+CD5+ | 207 |
| **B1a.PC.3** | B-1a (peritonial cavity) | CD19+B220+IgM++AA4-CD23-CD43+CD5+ | 202 |
| **Human ortholog genes shared by all B1a samples** | | | **125** |
| **B1b.PC.1** | B-1b (peritonial cavity) | CD19+B220+IgM++AA4-CD23-CD43+CD5- | 188 |
| **B1b.PC.2** | B-1b (peritonial cavity) | CD19+B220+IgM++AA4-CD23-CD43+CD5- | 199 |
| **B1b.PC.3** | B-1b (peritonial cavity) | CD19+B220+IgM++AA4-CD23-CD43+CD5- | 200 |
| **Human ortholog genes shared by all B1b samples** | | | **173** |
| **B.Fo.Sp.1** | B_Fo (follicular B lymphocytes) (spleen) | CD19+CD45R+IgM++AA4-CD23+CD43-CD50 | 163 |
| **B.Fo.Sp.2** | B_Fo (follicular B lymphocytes) (spleen) | CD19+CD45R+IgM++AA4-CD23+CD43-CD5- | 156 |
| **B.Fo.Sp.3** | B_Fo (follicular B lymphocytes) (spleen) | CD19+CD45R+IgM++AA4-CD23+CD43-CD5- | 156 |
| **Human ortholog genes shared by all B_Fo samples** | | | **140** |
| **B.MZ.Sp.1** | B_MZ (marginal zone B lymphocytes) | CD19+B220+IgM++AA4-CD23-CD21/35++ | 189 |
| **B.MZ.Sp.2** | B_MZ (marginal zone B lymphocytes) | CD19+B220+IgM++AA4-CD23-CD21/35++ | 194 |
| **B.MZ.Sp.3** | B_MZ (marginal zone B lymphocytes) | CD19+B220+IgM++AA4-CD23-CD21/35++ | 194 |
| **Human ortholog genes shared by all B_MZ samples** | | | **176** |
| **T.4Mem.Sp.1** | Memory CD4+ T cells (spleen) | CD4+ CD8- TCR+ CD25- CD44hi CD122Lo | 104 |
| **T.4Mem.Sp.4** | Memory CD4+ T cells (spleen) | CD4+ CD8- TCR+ CD25- CD44hi CD122Lo | 103 |
| **T.4Mem.Sp.5** | Memory CD4+ T cells (spleen) | CD4+ CD8- TCR+ CD25- CD44hi CD122Lo | 106 |
| **T.4Mem.LN.1** | Memory CD4+ T cells (subcutaneous lymph node) | CD4+ CD8- TCR+ CD25- CD44hi CD122Lo | 71 |
| **T.4Mem.LN.2** | Memory CD4+ T cells (subcutaneous lymph node) | CD4+ CD8- TCR+ CD25- CD44hi CD122Lo | 85 |
| **T.4Mem.LN.3** | Memory CD4+ T cells (subcutaneous lymph node) | CD4+ CD8- TCR+ CD25- CD44hi CD122Lo | 72 |
| **Human ortholog genes shared by all memory CD4+ T cells samples** | | | **48** |
| **T.4Nve.Sp.1** | CD4+ naïve T cells (spleen) | CD4+CD 8- TCR+ CD25- CD62Lhi CD44lo | 69 |
| **T.4Nve.Sp.2** | CD4+ naïve T cells (spleen) | CD4+CD 8- TCR+ CD25- CD62Lhi CD44lo | 60 |
| **T.4Nve.MLN.1** | CD4+ naive T cells (mesenteric lymph node) | CD4+CD 8- TCR+ CD25- CD62Lhi CD44lo | 63 |
| **T.4Nve.MLN.2** | CD4+ naive T cells (mesenteric lymph node) | CD4+CD 8- TCR+ CD25- CD62Lhi CD44lo | 61 |
| **T.4Nve.MLN.3** | CD4+ naive T cells (mesenteric lymph node) | CD4+CD 8- TCR+ CD25- CD62Lhi CD44lo | 53 |
| **T.4Nve.LN.1** | CD4+ naive T cells (subcutaneous lymph node) | CD4+CD 8- TCR+ CD25- CD62Lhi CD44lo | 70 |
| **T.4Nve.LN.2** | CD4+ naive T cells (subcutaneous lymph node) | CD4+CD 8- TCR+ CD25- CD62Lhi CD44lo | 62 |
| **T.4Nve.LN.3** | CD4+ naive T cells (subcutaneous lymph node) | CD4+CD 8- TCR+ CD25- CD62Lhi CD44lo | 63 |
| **T.4Nve.PP.1** | CD4+ naive T cells (peyer patches) | CD4+CD 8- TCR+ CD25- CD62Lhi CD44lo | 64 |
| **T.4Nve.PP.2** | CD4+ naive T cells (peyer patches) | CD4+CD 8- TCR+ CD25- CD62Lhi CD44lo | 55 |
| **Human ortholog genes shared by all CD4+ naïve T cells samples** | | | **42** |
| **T.8Mem.Sp.1** | Memory CD8+ T cells (spleen) | CD4-CD8+ TCR+ CD25- CD44hi CD122hi | 80 |
| **T.8Mem.Sp.3** | Memory CD8+ T cells (spleen) | CD4-CD8+ TCR+ CD25- CD44hi CD122hi | 78 |
| **T.8Mem.Sp.4** | Memory CD8+ T cells (spleen) | CD4-CD8+ TCR+ CD25- CD44hi CD122hi | 82 |
| **T.8Mem.LN.1** | Memory CD8+ T cells (subcutaneous lymph node) | CD4-CD8+ TCR+ CD25- CD44hi CD122hi | 61 |
| **T.8Mem.LN.2** | Memory CD8+ T cells (subcutaneous lymph node) | CD4-CD8+ TCR+ CD25- CD44hi CD122hi | 62 |
| **T.8Mem.LN.3** | Memory CD8+ T cells (subcutaneous lymph node) | CD4-CD8+ TCR+ CD25- CD44hi CD122hi | 63 |
| **Human ortholog genes shared by all memory CD8+ T cells samples** | | | **51** |
| **T.8Nve.Sp.1** | CD8+ naive T cells (spleen) | CD4- CD8+ TCR+ CD25- CD62Lhi CD44lo | 64 |
| **T.8Nve.Sp.2** | CD8+ naive T cells (spleen) | CD4- CD8+ TCR+ CD25- CD62Lhi CD44lo | 64 |
| **T.8Nve.Sp.3** | CD8+ naive T cells (spleen) | CD4- CD8+ TCR+ CD25- CD62Lhi CD44lo | 68 |
| **T.8Nve.MLN.1** | CD8+ naive T cells (mesenteric lymph node) | CD4- CD8+ TCR+ CD25- CD62Lhi CD44lo | 69 |
| **T.8Nve.MLN.3** | CD8+ naive T cells (mesenteric lymph node) | CD4- CD8+ TCR+ CD25- CD62Lhi CD44lo | 77 |
| **T.8Nve.MLN.4** | CD8+ naive T cells (mesenteric lymph node) | CD4- CD8+ TCR+ CD25- CD62Lhi CD44lo | 67 |
| **T.8Nve.LN.1** | CD8+ naive T cells (subcutaneous lymph node) | CD4- CD8+ TCR+ CD25- CD62Lhi CD44lo | 64 |
| **T.8Nve.LN.2** | CD8+ naive T cells (subcutaneous lymph node) | CD4- CD8+ TCR+ CD25- CD62Lhi CD44lo | 74 |
| **T.8Nve.LN.3** | CD8+ naive T cells (subcutaneous lymph node) | CD4- CD8+ TCR+ CD25- CD62Lhi CD44lo | 65 |
| **T.8Nve.PP.2** | CD8+ naïve T cells (Peyer patches) | CD4- CD8+ TCR+ CD25- CD62Lhi CD44lo | 65 |
| **Human ortholog genes shared by all CD8+ naïve T cells samples** | | | **46** |
| **DC.103.11b..Lv.1** | Dendritic cells (liver) | CD45+ CD11c+ MHC-II+ CD11b low CD103+ | 336 |
| **DC.103.11b..Lv.2** | Dendritic cells (liver) | CD45+ CD11c+ MHC-II+ CD11b low CD103+ | 330 |
| **DC.103.11b..Lv.1.1** | Dendritic cells (liver) | CD45+ CD11c+ MHC-II+ CD11b low CD103+ | 441 |
| **DC.103.11b..Lv.2.1** | Dendritic cells (liver) | CD45+ CD11c+ MHC-II+ CD11b low CD103+ | 471 |
| **DC.103.11b..Lv.3** | Dendritic cells (liver) | CD45+ CD11c+ MHC-II+ CD11b low CD103+ | 622 |
| **DC.103.11b..Lu.1** | Dendritic cells (lung) | CD11c+ CD8a- CD11blo CD103+ | 422 |
| **DC.103.11b..Lu.2** | Dendritic cells (lung) | CD11c+ CD8a- CD11blo CD103+ | 407 |
| **DC.103.11b..Lu.3** | Dendritic cells (lung) | CD11c+ CD8a- CD11blo CD103+ | 440 |
| **Human ortholog genes shared by all dendritic cells samples** | | | **122** |
| **NKT.44.NK1.1..Th.1** | Precursor iNKT | TCRbeta+ aGalCer tet+ CD44+NK1.1+ | 126 |
| **NKT.44.NK1.1..Th.1.1** | Precursor iNKT | TCRbeta+ aGalCer tet+ CD44+NK1.1+ | 126 |
| **NKT.44.NK1.1..Th.1.2** | Precursor iNKT | TCRbeta+ aGalCer tet+ CD44+NK1.1+ | 86 |
| **NKT.44.NK1.1..Th.2** | Precursor iNKT | TCRbeta+ aGalCer tet+ CD44+NK1.1+ | 119 |
| **NKT.44.NK1.1..Th.2.1** | Precursor iNKT | TCRbeta+ aGalCer tet+ CD44+NK1.1+ | 131 |
| **NKT.44.NK1.1..Th.2.2** | Precursor iNKT | TCRbeta+ aGalCer tet+ CD44+NK1.1+ | 116 |
| **NKT.44.NK1.1..Th.3** | Precursor iNKT | TCRbeta+ aGalCer tet+ CD44+NK1.1+ | 154 |
| **NKT.44.NK1.1..Th.4** | Precursor iNKT | TCRbeta+ aGalCer tet+ CD44+NK1.1+ | 140 |
| **Human ortholog genes shared by all iNKT samples** | | | **41** |
| **T.4FP3.25..Sp.2** | Tregs CD25+ Foxp3+ (spleen) | CD4+ CD8- TCR+ GFP+ CD25+ | 89 |
| **T.4FP3.25..Sp.3** | Tregs CD25+ Foxp3+ (spleen) | CD4+ CD8- TCR+ GFP+ CD25+ | 91 |
| **Human ortholog genes shared by all Treg samples** | | | **78** |
| **MF.Lu.1** | Macrophage | CD11chi CD11b- CD103-MHCII- SiglecF+ | 550 |
| **MF.Lu.2** | Macrophage | CD11chi CD11b- CD103-MHCII- SiglecF+ | 558 |
| **MF.Lu.3** | Macrophage | CD11chi CD11b- CD103-MHCII- SiglecF+ | 556 |
| **Human ortholog genes shared by all macrophages samples** | | | **497** |

Compilation of the immune cells samples and the number of genes obtained after analysis of microarrays data offered by ImmGen project. The signatures obtained were used to create gene sets in module map analysis.
